# Cannabinoids and Innate Immunity: Taking a Toll on Neuroinflammation

**DOI:** 10.1100/tsw.2011.84

**Published:** 2011-04-05

**Authors:** Eric J. Downer

**Affiliations:** Institute of Immunology, National University of Ireland Maynooth, County Kildare, Ireland

**Keywords:** cannabinoid, neuroinflammatory disorders, innate immune system, Toll-like receptors

## Abstract

The biologically active components of cannabis have therapeutic potential in neuroinflammatory disorders due to their anti-inflammatory propensity. Cannabinoids influence immune function in both the peripheral and the central nervous system (CNS), and the components of the cannabinoid system, the cannabinoid receptors and their endogenous ligands (endocannabinoids), have been detected on immune cells as well as in brain glia. Neuroinflammation is the complex innate immune response of neural tissue to control infection and eliminate pathogens, and Toll-like receptors (TLRs), a major family of pattern recognition receptors (PRRs) that mediate innate immunity, have emerged as players in the neuroinflammatory processes underpinning various CNS diseases. This review will highlight evidence that cannabinoids interact with the immune system by impacting TLR-mediated signaling events, which may provide cues for devising novel therapeutic approaches for cannabinoid ligands.

## NEUROINFLAMMATION

Inflammation in the central nervous system (CNS) (neuroinflammation) incorporates a spectrum of cellular processes that include the activation of glial cells, modulation of cytokine and chemokine balance, neuronal dysfunction, and neurodegeneration[1]. Indeed, neuroinflammatory events are integral to disorders such as Alzheimer's disease (AD), Parkinson's disease (PD), multiple sclerosis (MS), and stroke[2]. Immune surveillance occurs in the CNS, dispelling previous dogma that the CNS is an immune-privileged site, and such immune control directs the neuroinflammatory response in neurological disorders[3]. The classical paradigm associates neuroinflammation with strong infiltration of the CNS with proinflammatory blood leukocytes due to blood brain barrier (BBB) alterations[4]. Leukocyte arrival in the CNS is followed by production of reactive oxygen species, proinflammatory cytokines, and cytolytic enzymes, leading to detrimental effects on neuronal functioning. However, long-standing investigations have also identified glial cells, particularly microglia, as key players in both acute and chronic neurological conditions[5]. Upon CNS injury, microglia transform to an "activated" form, whereby they display a phagocytic and ramified phenotype, participating in disease pathogenesis via the production of oxygen radicals, cytokines, glutamate, and proteases[6]. The activity of microglia is controlled by multiple factors, including interferon (IFN)-γ, a T-cell cytokine, and β-amyloid (Aβ), in addition to their complex interaction with neurons, astrocytes, and T cells[7].

Toll-like receptors (TLRs) are type-I transmembrane glycoproteins involved in the recognition of conserved microbial motifs[8]. They continue to emerge as key players in infectious and noninfectious CNS diseases, in addition to their defined role in innate immunity (see below). TLR-mediated responses can be beneficial or detrimental, depending on the strength and timing of the activating signal. With respect to neuroinflammatory conditions, specific roles of TLRs have been shown in animal disease models[9,10], while the expression of TLRs, and their related signaling proteins, has been characterized in CNS glia and neurons[11]. Hence, while current treatments for neurological conditions are based on regulating inflammation and neurotransmission, in addition to immunomodulation and repair of damage, modulating the innate immune response by targeting TLRs and their signaling cascades in the CNS may represent a strong therapeutic avenue for neuroinflammatory conditions.

## INNATE IMMUNE SIGNALING IN THE CNS

The innate immune system represents the earliest defense against pathogens, providing protection against infections and self-antigens. This system is orchestrated by a number of cells, including mast cells, dendritic cells (DCs), neutrophils, natural killer (NK) cells, γ δ T cells, and macrophages. These cells act as crucial initiators/effectors of innate immune responses, and play diverse roles in the pathogenesis of neuroinflammatory conditions via the secretion of cytokines, activation and differentiation of naïve T cells, and production of superoxide and other reactive oxygen species[12]. Furthermore, in the last 15 years, it has become clear that glial cells play an active role in the CNS[7] and this role in innate immune responses in the CNS is central to this review.

Innate immunity is tightly regulated by a complex mechanism involving pattern recognition receptors (PRRs) that recognize molecular signatures of microbes known as pathogen-associated molecular patterns (PAMPs). Intracellular signaling triggered by PRRs leads to transcriptional expression of inflammatory mediators that coordinate the elimination of pathogens and infected cells[13]. PRRs are expressed on innate immune cells and are involved in microbe recognition/internalization by phagocytes (endocytic receptors), cell activation in response to diverse microbial moieties (signaling receptors), and apoptotic cell recognition (bridging molecules and endocytic receptors)[14]. TLRs belong to the family of signaling PRRs, along with retinoic acid–inducible gene I (RIG-I)–like receptors (RLRs) and nucleotide-binding oligomerization domain (NOD)–like receptors (NLRs)[14]. If left unchecked, or not tightly regulated, dysregulation of this system can lead to conditions such as sepsis, asthma, and autoimmunity[15]. NLRs respond to pathogens to activate pro- and anti-inflammatory pathways, with mutations or polymorphisms in both human or mouse NLRs linking these receptors with inflammatory disorders, such as Crohn's disease[16]. Similarly, some evidence, although limited, demonstrates that RLRs can activate inflammatory cascades that may have consequences for the progression of neuroinflammatory responses[17].

TLR activation is a major inducer of neuroinflammation, with a large body of evidence demonstrating that TLR ligands induce CNS inflammation via production of cytokines, nitric oxide, and chemokines[15,18]. The extracellular domains of TLRs contain leucine-rich repeat motifs for recognition of their respective pathogens and a conserved intracellular Toll-interleukin-1 receptor (TIR) domain to initiate downstream signaling[8]. TLRs are involved in the recognition of bacterial/viral products by the activation of transcription factors, such as nuclear factor (NF)-κB, and the subsequent induction of genes encoding IFNs and proinflammatory cytokines[8]. To date, 13 mammalian TLRs have been identified and, with the exception of TLR3, all TLRs recruit the adaptor myeloid differentiation factor 88 (MyD88)[19] (Fig. 1). TLR3 induces MyD88-independent signaling to regulate NF-κB via the TIR-domain–containing adaptor-inducing IFN-β (TRIF) adaptor protein. Such TRIF-mediated signaling constitutes the MyD88-independent pathway and, in addition to stimulating NF-κB, this pathway promotes

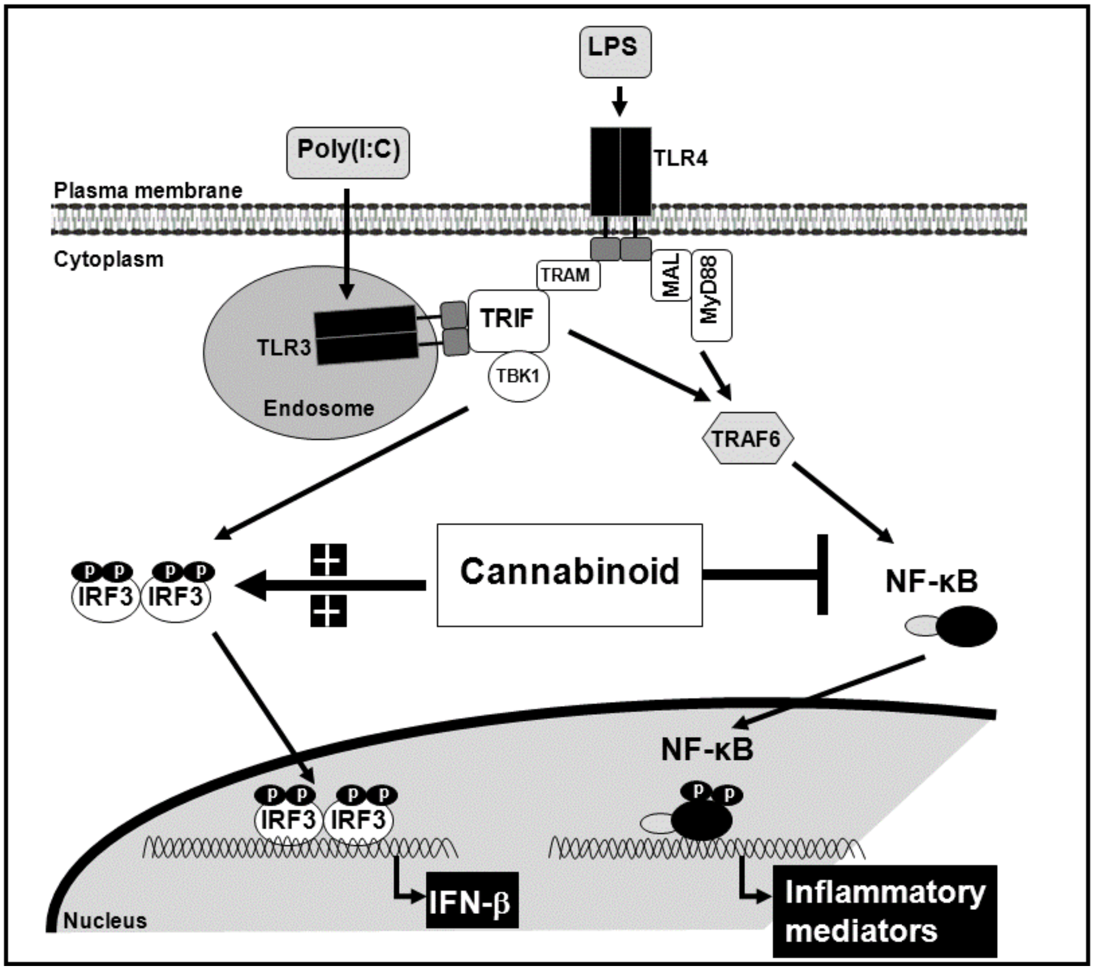

**FIGURE 1.** Mechanism of *R*(+)WIN55,212-2-induced regulation of TLR3 and TLR4 signaling[68]. *R*(+)WIN55,212-2 inhibits the proinflammatory signaling axis triggered by TLR3 and TLR4, while selectively augmenting TLR3-induced expression of IFN-β. Innate immune recognition by TLRs culminates in the induction of an array of immune response genes, including proinflammatory cytokines and chemokines, in addition to type-I IFNs. TLRs induce signaling via recruitment of the adaptor MyD88, with the exception of TLR3, which induces Myd88-independent signaling via TRIF protein. TRIF-mediated signaling promotes the phosphorylation and nuclear localization of transcription factor IRF3 and subsequent induction of type-I IFNs. TLR4 uses Myd88-dependent and -independent pathways to activate NF-κB via the downstream adaptor tumor necrosis factor (TNF) receptor-associated factor 6 (TRAF-6), resulting in the translocation of an active heterodimer protein to the nucleus and induction of proinflammatory genes.

the phosphorylation of another family of transcription factors, the IFN regulatory factors (IRFs)[20]. The phosphorylation of IRFs promotes their nuclear translocation and induction of type-I IFNs[8]. TLR4 can initiate both MyD88- and TRIF-dependent signaling pathways, but can only recruit these adaptors by first employing the bridging adaptors, MyD88 adaptor-like (Mal) and TRIF-regulated adaptor molecule (TRAM)[8] (Fig. 1). Hence, overstimulation of TLR pathways can lead to increased levels of type-I IFNs and proinflammatory cytokines that exacerbate inflammation in the CNS.

While early reports on TLR expression patterns focused mainly on immune cells (DCs, T cells, macrophages)[21] and their role in orchestrating a comprehensive program to remove invading pathogens, it has become increasingly clear that TLRs have a distinct role in immune surveillance and inflammatory responses in the CNS. Indeed, more recent studies have characterized TLR expression profiles on resident CNS cells, including microglia (TLRs 1–9)[22], astrocytes (TLRs 1–5, 9, 13)[23,24,25], and neurons (TLRs 2–5, 8, 9, 11–13)[24,26,27]. TLR expression has been determined on glial cells in human CNS tissue derived from patients suffering from MS and other diseases, and interestingly, changes in the TLR expression profile have been determined in the neuroinflammed brain[28]. Knockout studies have elucidated the complex role of TLRs and TLR signaling proteins in neuroinflammatory conditions. Indeed, TLR2[18], TLR9[29], and MyD88[29] deficiency is protective in models of neuroinflammation, while TLR4[30], TLR2[31], and TRIF (receptor proximal adaptor for TLR3 and TLR4)[32] deficiency has been shown to exacerbate disease in neuroinflammatory models, indicating the complex interplay of these pathways in inflammatory conditions.

## CANNABINOID CHARACTERISTICS

Cannabinoids incorporate the components of the cannabis plant (*Cannabis sativa L.*), the endogenous cannabinoids (endocannabinoids), and the synthetic cannabinoid ligands[33]. The cannabinoid system is linked with all aspects of human physiology and the biological effects of cannabis intake include the impairment of short-term memory, acute panic reactions, and motor incoordination[34]. Cannabinoids elicit diverse central and peripheral effects by activating G protein–coupled cannabinoid receptors $CB_1$ and $CB_2$, the expression of which has been localized on glia[35], immune cells[36], and neurons[37]. Indeed, much experimental evidence indicates that cannabinoid receptors are key players in mediating neuroinflammatory events[38,39]. $CB_1$ receptors are expressed at high levels throughout the brain[40], while $CB_2$ receptor expression is predominantly restricted to glia, with neuronal $CB_2$ expression restricted to a population of neurons in the brainstem[41]. Importantly in microglia, changes in $CB_1$ and $CB_2$ expression have been determined depending on their activation profile, with $CB_2$ expression up-regulated on activated microglia in CNS tissue in response to neuroinflammatory events[42], although the implications of this are not clear. Studies in $CB_1$, $CB_2$, or double-knockout mice have revealed non–$CB_1$/$CB_2$ receptor-mediated cannabinoid effects[43] with potential candidates identified as the orphan G protein–coupled receptor GPR55[44], transient receptor potential vanilloid type-1 (TRPV1) channel[45], or the nuclear receptor superfamily of peroxisome proliferator-activated receptors (PPARs)[46]. Following cannabinoid receptor interaction, cannabinoids couple to multiple signal transduction pathways, including mitogen-activated protein (MAP) kinases, adenylyl cyclase, and ion channels, with the cellular outcome depending on both the cell type and the cell context[47].

Several therapeutic effects have been ascribed to cannabis use, including the alleviation of intraocular pressure[48] and emesis[49]. To date, three cannabis-based medicines are in the clinic, including Cesamet® (nabilone; a synthetic derivative of the plant-derived cannabinoid tetrahydrocannabinol; THC), Marinol® (dronabinol; synthetic THC), and Sativex® (a combination of two plant-derived cannabinoids, THC and cannabidiol [CBD]). Cesamet and Marinol are used as antiemetics following chemotherapy, while Sativex has demonstrated efficacy in MS patients with central pain and spasticity[50]. Furthermore, the neuroprotective[51], anti-inflammatory (see below)[52], and antitumoral[53] propensity of cannabinoids make them promising therapeutic targets.

## CANNABINOID ROLE IN NEUROINFLAMMATION

Cannabinoids are currently under investigation for the treatment or management of inflammatory conditions, including MS, rheumatoid arthritis, AD, and glaucoma[52]. In particular, it is accepted that some MS patients self-medicate with cannabis, which is suggested by anecdotal evidence to be beneficial in controlling symptoms such as pain, spasticity, and tremor, and is supported by clinical trials of medical cannabis extracts[54]. Such studies paved the way for clinical studies on Sativex, an oromucosal pump spray containing a combined cannabinoid medicine constituted by the two plant-derived cannabinoids

THC and CBD in a 1:1 ratio[55]. Sativex was developed by GW Pharmaceuticals and is currently prescribed for the neuropathic pain and spasticity associated with MS, in addition to cancer pain[50]. It is noteworthy that beneficial effects of cannabinoid agonists have been demonstrated in PD patients[56], with further trials revealing beneficial effects of cannabinoids in AD patients[57]. Further studies will elucidate the clinical potential of cannabinoid compounds in these conditions.

Research evidence demonstrating the anti-inflammatory properties of cannabinoids in animal models of neuroinflammation indicates that cannabinoid administration attenuates the clinical development of experimental autoimmune encephalomyelitis (EAE), an animal model of MS[58], in addition to exerting anti-inflammatory properties in rodent models of AD-related neuroinflammation[59], PD[60], head injury[61], aging[62], and ischemia[63]. Much *in vitro* data support these findings. Indeed, protective effects of cannabinoids have been demonstrated against IL-1β–[64], IFN-γ–[65], Aβ-[66], hypoxia-ischemia–[67], and TLR4- (see below)[68] induced inflammation in cultured CNS cells. In addition, evidence from knockout studies indicates cannabinoid receptor involvement in the neurodegenerative and neuroinflammatory pathology associated with EAE[39], closed head injury[69], and cerebral ischemia[70]. In support of this, it has been demonstrated that endogenous cannabinoid concentration is altered in the cerebrospinal fluid and peripheral lymphocytes of relapsing MS patients, and in the brain of EAE mice[71]. Furthermore, such findings are not restricted to MS. Hence, increased endocannabinoid content has been detected in the basal ganglia of rats in a model of PD[72], in peripheral blood from stroke patients[73], in ischemic brain regions in murine stroke models[74], and in a murine closed head injury model[75]. The endocannabinoid system has emerged as a key player in pathophysiological mechanisms, and the plasticity of this system in neuroinflammatory and neurodegenerative disorders, such as the time- and brain region–specific changes in cannabinoid receptors and the enzymes that synthesize and hydrolyze endocannabinoids, offers both therapeutic avenues for manipulation and insights into the immunomodulatory role of this system in disorders such as AD, MS, and PD[76].

## CANNABINOID EFFECTS ON INNATE IMMUNE CELLS

TLRs are central in the innate immune and neuroimmune response, and evidence indicates that TLR activation is a major inducer of neuroinflammation[15]. Cannabinoid receptors have been localized on the major glial cells[35] and a large body of evidence demonstrates that cannabinoids can negatively regulate TLR4-induced inflammation in these cells. Indeed, in microglia, THC[77], CBD[78], endocannabinoids (anandamide and 2-arachidonoylglycerol; 2-AG)[79], and the synthetic cannabinoid receptor agonists *R*(+)WIN55,212-2, methanandamide, and CP55,940[79,80,81,82], ablate proinflammatory mediator production induced by the TLR4 agonist lipopolysaccharide (LPS), a component of the outer membrane of Gram-negative bacteria. In parallel, findings elsewhere demonstrate that anandamide[83], CP55,940[83], and *R*(+)WIN55,212-2[64,68] attenuate TLR4-induced neuroinflammatory changes in astrocytes. Furthermore, in mixed glial cultures, synthetic cannabinoids HU210 and CP55,940 enhance TLR4-induction of the anti-inflammatory cytokine IL-1 receptor antagonist[84], while *R*(+)WIN55,212-2 and the plant-derived cannabinoid cannabinol (CBN) inhibit TLR4-induced nitric oxide production in glioma cells[85]. These data are supported by *in vivo* evidence showing that HU210 prevents the accumulation of proinflammatory cytokines in the rat brain following intraperitoneal administration of LPS[86], while *R*(+)WIN55,212-2 exerts a similar effect following central administration of LPS to the CNS[87]. TLRs and cannabinoid receptors share some common signaling components (e.g., MAP kinases). However, while evidence indicates cannabinoid receptor dependency[83] and independency[68] for cannabinoid effects on TLR-induced signaling events, no direct cross-talk between these cascades has been determined to date.

We have demonstrated recently that *R*(+)WIN55,212-2, in addition to acting as a novel regulator of TLR3 signaling, can also impact TLR4-induced proinflammatory signaling cascades[68]. Specifically, our findings indicate that *R*(+)WIN55,212-2 impacts TLR signaling by inhibiting the proinflammatory signaling axis triggered by TLR3 and TLR4, while selectively augmenting TLR3-induced expression of

IFN-β[68], a type-I IFN currently used as a first-line treatment of MS[88] (Fig. 1). The mechanism(s) of action of IFN-β is complex with demonstrated effects on antigen presentation, costimulatory molecule expression, T-cell proliferation, and leukocyte migration[89]. Elsewhere, Kozela and colleagues[90] demonstrate that THC and CBD inhibit TLR4-induced proinflammatory signaling and production of IFN-β expression in microglia, but failed to address the specific impact of these cannabinoids on TLR3-induced cascades triggered by selective TLR3 agonists. Additional data are required to further elucidate the regulatory role of cannabinoids on other TLR cascades.

Cells of the innate immune system are pivotal players in neuroinflammatory conditions, and immunotherapeutic intervention by targeting these cells offers an attractive treatment strategy. In addition to CNS cells, cannabinoid receptors have been localized on virtually all immune cells associated with neuroinflammation, including NK cells, DCs, neutrophils, and macrophages[36]. In macrophages, which play an important role in innate and adaptive immunity, *R*(+)WIN55,212-2[91], CP55,940[92], THC[93], and anandamide[94] inhibit TLR4-induced inflammation in a manner similar to that observed by cannabinoids in glia. In addition, THC induces apoptosis in macrophages[95], while THC and CP55,940 can inhibit macrophage migration[96]. Elsewhere, data indicate that *R*(+)WIN55,212-2[97], anandamide[98], and CBD[99] inhibit neutrophil migration, a key event in brain injury[100]. Furthermore, THC[101] and CP55,940[102] negatively regulate NK cell activity, an event possibly associated with cannabinoid-induced immunosuppression. It is well recognized that cannabinoids further target DC function. Indeed, THC induces cell death in DCs[103], while anandamide has been shown to reduce DC capacity to stimulate T cells[104], in addition to inducing an increment in DC activation[105]. Interestingly, JWH 133, a selective $CB_2$ agonist, suppresses LPS-induced TLR4 and MyD88 up-regulation in DCs[106], which may contribute to the anti-inflammatory effect of this cannabinoid in autoimmune disease.

Given the immunoregulatory properties of cannabinoids, it is important to note that cannabis users may display impaired immunological functions. Indeed, cannabis use has been associated with a predisposition to development of opportunistic infections, bacterial pneumonia[107], and more rapid progression of HIV in AIDs patients[108]. This is supported by animal data demonstrating cannabinoid-induced suppression of cytokine production[109] and enhanced progression of HIV infection[110]. Consideration of cannabinoid therapies as anti-inflammatory agents should be weighed in light of their potential effects in all aspects of the immune system, with further in-depth research on cannabinoid impact on innate and adaptive arms of the immune system delineating the clinical promise of cannabinoids as anti-inflammatory agents.

## CONCLUDING REMARKS

Innate immune responses in the CNS are pivotal in the elimination of pathogens and promotion of cell survival. However, dysregulation of this system is associated with neuroinflammation and degeneration. TLRs, in addition to their role in innate immune control, have emerged as central players in neuroinflammation, ensuring that TLR agonists/antagonists are currently undergoing preclinical and clinical evaluation for the treatment of sepsis and inflammatory disease. Cannabinoids are recognized as anti-inflammatory agents, with identified protective roles in many neuroinflammatory disorders. This review summarized the main data demonstrating the propensity of cannabinoid compounds to impact TLR-induced signaling, further highlighting the potential of these compounds to regulate innate immune cell functioning and subsequent inflammatory events. Although these cannabinoid effects await further in-depth studies, it is clear that cannabinoid impact on the TLR system presents a pharmacological target with promising therapeutic potential.

## ACKNOWLEDGMENTS

This publication emanated from research funded by an Embark Initiative (Irish Research Council for Science, Engineering and Technology) fellowship. The author is grateful to Dr. Janis Noonan, Dr. Mark Mellett, and Dr. Paola Atzei for helpful discussions on the manuscript.
